# Autophagic Induction Greatly Enhances *Leishmania major* Intracellular Survival Compared to *Leishmania amazonensis* in CBA/j-Infected Macrophages

**DOI:** 10.3389/fmicb.2018.01890

**Published:** 2018-08-15

**Authors:** Beatriz R. S. Dias, Carina S. de Souza, Niara de Jesus Almeida, José G. B. Lima, Kiyoshi F. Fukutani, Thiale B. S. dos Santos, Jaqueline França-Cost, Claudia I. Brodskyn, Juliana P. B. de Menezes, Maria I. Colombo, Patricia S. T. Veras

**Affiliations:** ^1^Laboratory of Parasite-Host Interaction and Epidemiology, Gonçalo Moniz Institute, Salvador, Brazil; ^2^Laboratory of Inflammation and Biomarkers, Gonçalo Moniz Institute, Salvador, Brazil; ^3^Department of Biointeraction, Federal University of Bahia, Salvador, Brazil; ^4^Laboratory of Cellular and Molecular Biology, Institute of Histology and Embryology-CONICET, National University of Cuyo, Mendoza, Argentina

**Keywords:** *Leishmania*, macrophages, autophagy, LC3, parasitophorous vacuole

## Abstract

CBA mouse macrophages control *Leishmania major* infection yet are permissive to *Leishmania amazonensis*. Few studies have been conducted to assess the role played by autophagy in *Leishmania* infection. Therefore, we assessed whether the autophagic response of infected macrophages may account for the differential behavior of these two parasite strains. After 24 h of infection, the LC3-II/Act ratio increased in both *L. amazonensis*- and *L. major*-infected macrophages compared to uninfected controls, but less than in chloroquine-treated cells. This suggests that *L. amazonensis* and *L. major* activate autophagy in infected macrophages, without altering the autophagic flux. Furthermore, *L. major-*infected cells exhibited higher percentages of DQ-BSA-labeled parasitophorous vacuoles (50%) than those infected by *L. amazonensis* (25%). However, *L. major*- and *L. amazonensis*-induced parasitophorous vacuoles accumulated LysoTracker similarly, indicating that the acidity in both compartment was equivalent. At as early as 30 min, endogenous LC3 was recruited to both *L. amazonensis*- and *L. major*-induced parasitophorous vacuoles, while after 24 h a greater percentage of LC3 positive vacuoles was observed in *L. amazonensis*-infected cells (42.36%) compared to those infected by *L. major* (18.10%). Noteworthy, principal component analysis (PCA) and an hierarchical cluster analysis completely discriminated *L. major*-infected macrophages from *L. amazonensis*-infected cells accordingly to infection intensity and autophagic features of parasite-induced vacuoles. Then, we evaluated whether the modulation of autophagy exerted an influence on parasite infection in macrophages. No significant changes were observed in both infection rate or parasite load in macrophages treated with the autophagic inhibitors wortmannin, chloroquine or VPS34-IN1, as well as with the autophagic inducers rapamycin or physiological starvation, in comparison to untreated control cells. Interestingly, both autophagic inducers enhanced intracellular *L. amazonensis* and *L. major* viability, while the pharmacological inhibition of autophagy exerted no effects on intracellular parasite viability. We also demonstrated that autophagy induction reduced NO production by *L. amazonensis*- and *L. major*-infected macrophages but not alters arginase activity. These findings provide evidence that although *L. amazonensis*-induced parasitophorous vacuoles recruit LC3 more markedly, *L. amazonensis* and *L. major* similarly activate the autophagic pathway in CBA macrophages. Interestingly, the exogenous induction of autophagy favors *L. major* intracellular viability to a greater extent than *L. amazonensis* related to a reduction in the levels of NO.

## Introduction

Leishmaniasis, a disease caused by trypanosomatids of the genus *Leishmania*, represents an emerging disease that continues to present a major public health problem, especially due to elevated incidence in developing countries (Santos et al., 2008; Perinoto et al., 2010). According to the World Health Organization, around 15 million people are affected worldwide in 98 countries, including 72 developing nations (World Health Organization, 2010; Alvar et al., 2012). *Leishmania* spp. are obligate intracellular parasites that live and multiply within modified phagolysosome compartments, denominated parasitophorous vacuoles, in macrophages, the main host cell in vertebrate hosts (Russell and Wilhelm, 1986).

Since the early 2000s, many fields of study have focused research efforts on the physiological process of autophagy. This evolutionarily conserved process is responsible for the degradation of organelles and proteins during cell differentiation and under stress conditions (Meijer and Codogno, 2004; Levine and Deretic, 2007; Duszenko et al., 2011). During the autophagic degradative process, cytosolic components become trapped into compartments called autophagosomes, which subsequently fuse with lysosomes to form autolysosomes in consecutively coordinated steps: initiation, nucleation, expansion, completion, docking and fusion, thereby resulting in intravacuolar cargo degradation. These events are dependent on the family of autophagy related genes (Atg) and proteins that play specific roles during each stage of the autophagic process (Suzuki and Ohsumi, 2007; Suzuki et al., 2010; Nazarko et al., 2011). The initiation step results in phagophore formation, which is mostly dependent on the assembling of the Beclin 1-Vps34 class III PI(3)K complex in autophagosomal membranes of differing origin (Levine and Deretic, 2007). Two conjugation systems can be subsequently recruited to support the expansion of the phagophore: the Atg12-Atg5-Atg16 complex, or the Atg8 protein conjugated to phosphoethalanolamine (PE), which is referred as LC3-II in mammals. This protein, central in the autophagic process, is generated by site-specific proteolysis and lipidation occurring near the C-terminus of the cytosolic protein Atg8 (LC3-I) (Lang et al., 1998). As LC3-II is integrated into the membranes of newly formed autophagosomes, levels of LC3-II correlate positively with numbers of cellular autophagosomes (Kabeya et al., 2000). Thus, LC3-II has been employed as a useful marker for studies investigating the dynamics of autophagic pathway activation.

In recent decades, the role played by autophagy in mammalian cells during infection arising from a variety of pathogens has been extensively investigated (Gutierrez et al., 2004; Levine and Deretic, 2007; Schnaith et al., 2007; Mestre et al., 2010). Several studies have implicated autophagic pathway activation in the triggering of an innate immune response, which results in the protection of host cells against infections arising from intracellular microorganisms, such as *Mycobacterium tuberculosis* and *Streptococcus pyogenes* (Gutierrez et al., 2004; Nakagawa et al., 2004; Gutierrez et al., 2005; Levine and Deretic, 2007). On the other hand, autophagic pathway activation can also favor the survival of some species of intracellular pathogens, such as *Staphylococcus aureus* and *Coxiella burnetti* (Gutierrez et al., 2005; Schnaith et al., 2007; Mestre et al., 2010).

To date, few studies have attempted to assess the role of autophagy in infection by parasites of the *Leishmania* genus. A seminal work by Schaible and colleagues demonstrated that large *L. mexicana-*induced parasitophorous vacuoles acquired macromolecules from the host cell cytoplasm via microautophagy (Schaible et al., 1999). These authors suggested that these macromolecules, once transferred from the host cell cytoplasm to the lumen of *L. mexicana*-induced parasitophorous vacuoles, seemed to be endocytosed by parasites (Russell et al., 1992). More recently, it has been demonstrated that both *L. amazonensis* and *L. major* infection induces autophagic pathway activation in susceptible BALB/c mouse macrophages (Cyrino et al., 2012; Frank et al., 2015). Investigations in the literature regarding the role played by autophagy in *Leishmania* infection have led to controversial data. Pinheiro et al. (2009) showed that in macrophages from susceptible BALB/c mice, but not in macrophages of *L. amazonensis*-resistant C57BL/6 mice, the autophagic induction resulted in increased intracellular load of *L. amazonensis* (Pinheiro et al., 2009). Also, these authors demonstrated that autophagy induced by starvation did not alter intracellular *L. major* parasitic load in susceptible BALB/c mouse macrophages (Pinheiro et al., 2009). Studies employing the genetic modification of autophagic-related genes have also presented inconclusive results. Thomas et al. (2017) demonstrated that Atg5 and Atg9 knockdown in the human monocytic cell-line, THP-1, reduced *L. donovani* survival, suggesting that autophagy is beneficial to *Leishmania* infection. This stands in contrast to BALB/c macrophages knocked-down for Atg5, which served to enhance *L. major* parasitic load (Frank et al., 2015). Consistent with these authors’ findings, another study recently showed that Atg5 knockdown in the *L. major*-resistant C57BL/6 macrophages resulted in increased parasite replication (Franco et al., 2017), which suggests that the activation of an Atg5-dependent autophagic process could result in the elimination of *L. major* intracellular parasites.

The CBA mouse model has demonstrated susceptibility to *L. amazonensis*, while controlling *L. major* infection *in vivo* (Lemos de Souza et al., 2000). Moreover, while CBA mouse macrophages were shown to be permissive to *L. amazonensis in vitro*, these same cells also reduced *L. major* infection (Gomes et al., 2003). Therefore, we addressed whether the autophagic response of infected macrophages may account for the differential behavior of these two parasite strains in CBA macrophages. Accordingly, the present study took advantage of the *in vitro* CBA infection model to comparatively characterize *L. major* and *L. amazonensis*-induced compartments with respect to autophagic features, in addition to investigating the effects of autophagic modulation on *Leishmania* infection outcome using a pharmacological approach.

## Materials and Methods

### Ethics Statement

The CBA mice used in the present study were provided by the animal care facility at the Gonçalo Moniz Institute - FIOCRUZ/BA, following approval by the Institutional Animal Experimentation Review Board (CEUA) under protocol number 015/2014. Animals were kept and handled in accordance with the norms recommended by the International Guiding Principles for Biomedical Research Involving Animals; all experimental protocols complied with these guidelines, as well as all resolutions established by the Brazilian National Council for the Control of Animal Experimentation (CONCEA).

All protocols, analytic methods and material used in the present study are available upon request to all interested researchers.

### Macrophage Culture

To obtain inflammatory peritoneal macrophages, 2.5 mL of 3% thioglycolate (Sigma, St. Louis, MO, United States) was injected into the peritoneal cavities of CBA mice, as previously described by Gomes et al. (2003). After 96 h, mice were euthanized, and peritoneal cavities were washed twice using 10 mL of 0.9% NaCl with heparin (20 U.I./mL) (Cristália, Itapira, SP, BR). Next, macrophages were centrifuged at 300 × g under 4°C for 10 min and plated in complete DMEM (Dulbecco’s modified Eagle medium) (Gibco, Grand Island, NY, United States) supplemented with 25 mM HEPES (N-2-hydroxyethyl piperazine-*N*’-2-ethane-sulfonic acid) (Sigma, St Louis, MO, United States) adjusted to pH 7.4, 2 mM glutamin (Gibco, Grand Island, NY, United States), 20 g/mL ciprofloxacin (Isofarma, Precabura, CE, BR) and 10% inactivated fetal bovine serum (Gibco, Grand Island, NY, United States), then incubated overnight at 37°C under 5% CO_2_ and 95% humidity.

### *Leishmania* Culturing

Axenic cultures of *L. amazonensis* (MHOM/Br88/Ba-125) or *L. major* (MHOM/RI/-/WR-173) promastigotes were maintained for up to six successive passages in Schneider’s Insect Medium (Sigma, St Louis, MO, United States) supplemented with 50 μg/mL gentamicin (Gibco, Grand Island, NY, United States) and 10% fetal bovine serum (Gibco, Grand Island, NY, United States), following a slightly modified previously described protocol (Gomes et al., 2003). Promastigotes were grown in an incubator at 24°C and monitored daily by counting in a Neubauer chamber. Upon reaching the stationary phase, the promastigotes were subjected to separation of infective metacyclic forms using a Ficoll-Paque gradient as described by Spath and Beverley (2001).

### Labeling of *L. amazonensis* and *L. major* Promastigotes With Carboxyfluorescein Succinimidyl Ester (CFSE)

For some experiments, *L. amazonensis* and *L. major* were labeled with CFSE (21888) (Sigma, St Louis, MO, United States) as described by Chang et al. (2007). Parasites (10^8^) were suspended in 2 mL of 0.9% NaCl solution containing 1 μM CFSE for 10 min at 37°C, protected from light. To stop the reaction, a similar volume (2 mL) of fetal bovine serum was added to each parasite suspension. After 1 min of incubation, parasite suspensions were washed twice with 0.9% NaCl solution and centrifuged at 1,800 × *g* for 10 min under 4°C. After counting with a Neubauer chamber, parasites were used at the experimental concentrations described below.

### Macrophage Infection With *L. amazonensis* or *L. major*

CBA mouse macrophages were plated in either 6-well plates (2 × 10^6^/2 mL) or 24-well plates (2 × 10^5^/mL) containing coverslips for 24 h at 37°C, and then infected with metacyclic promastigotes of *L. amazonensis* or *L. major* at a 5:1 (parasite:macrophage) ratio in accordance with one of the following protocols:

(i) macrophages were either infected for 30 min, or infected for 4 h followed by a 24 h-incubation period to evaluate autophagic activation and flux, as described below in **Evaluation of autophagic induction and flux in macrophages infected by *L. amazonensis* or *L. major***;
(ii) macrophages were either infected for 30 min or 4 h to characterize the autophagic features within *Leishmania* parasitophorous vacuoles at early stages of infection. All macrophages were then washed and fixed. Alternatively, to compare *L. amazonensis* and *L. major* parasitophorous vacuole characteristics at later stages of infection, another group of macrophages was infected for 4 h and then washed with saline to remove any non-internalized promastigotes. After an additional 24 h-incubation period, these macrophages were then washed and fixed. Autophagic features were then evaluated at both early and late stages of infection as described below in **Evaluation of hydrolytic activity in *L. amazonensis*- or *L. major*-induced parasitophorous vacuoles; LysoTracker labeling of *L. amazonensis* or *L. major* parasitophorous vacuoles and Evaluation of the recruitment of LC3-II to *L. amazonensis* or *L. major* parasitophorous vacuoles**;
(iii) macrophages were first infected for 30 min, treated with different autophagic modulators for an additional 4 h, then washed and reincubated in modulator-free medium as described below in **Modulation of the autophagic process in *L. amazonensis* or *L. major* infection**. Following each infection protocol, all plates were washed to remove non-internalized parasites. Finally, all plates containing cells infected with *L. amazonensis* were reincubated at 35°C, while those infected with *L. major* were incubated at 37°C, both supplemented with 5% CO_2_ and 95% humidity.

### Evaluation of Autophagic Induction and Flux in Macrophages Infected by *L. amazonensis* or *L. major*

For these experiments, western blotting was used to measure the ratio between the amount of membrane bound LC3-II and actin (Act) in extracts of CBA mouse macrophages. Cells were plated and infected in accordance with the protocol (i) described above in **Infection of macrophages with** ***L. amazonensis* or *L. major***. To evaluate autophagic flux, either uninfected or cells infected with *L. amazonensis* or *L. major* were treated with chloroquine (10 μM), a drug that blocks the fusion of autophagosomes with lysosomes (Klionsky et al., 2016), for an additional 4 h. Upon the completion of infection or treatment protocols, cells were scraped, recovered and centrifuged at 720 × *g* for 10 min under 4°C. Cells were then resuspended in RIPA buffer [1 M Tris HCl pH 8, 1 M NaCl, Substitute Nonidet P40 (Sigma, St Louis, MO, United States), 10% Sodium Dodecyl Sulfate (Riedel-de-Haen, Seelge, GER), 5% DOC, Mili-Q Water and Protease Inhibitor (Roche)] and incubated for 30 min at 4°C. Next, cellular extracts were sonicated 3x for 10 s at 127V in a Branson Sonifier apparatus. Samples were then centrifuged at 13,000 × *g* under 4°C for 20 min and quantified using a Nanodrop spectrophotometer (Thermo Scientific).

Initially, 60 μg of protein from each extract was subjected to electrophoresis in a Bio-Rad Mini-Protean Tetra System. After 1 h 30 min of gel running, proteins were transferred to a nitrocellulose membrane using a Bio-RAD Trans-blot Turbo apparatus for a period of 30 min at 25 V, 1A. The membrane was then blocked with 5% skim milk in PBST (1x PBS + 0.1% Tween 20) for 1 h at room temperature. Membranes were then incubated overnight at 4°C with anti-LC3 antibody (NB600-1384, Novus Biologicals, Littleton, CO, United States) or anti–actin (Sigma) diluted in PBST + 3% milk solution. Each membrane was subsequently incubated with HRP-coupled anti-rabbit IgG or anti-mouse IgG secondary antibodies. Blots were then developed with an ECL Chemiluminescence Kit (Thermo Fisher Scientific, Rockford, IL, United States) and detected using a Luminescent Image Analyzer and Image Quant Las 4000 software. Densitometry quantification of the bands was performed using Image J software.

### Evaluation of Hydrolytic Activity in *L. amazonensis*- or *L. major*-Induced Parasitophorous Vacuoles

Hydrolytic activity in the parasitophorous vacuoles within macrophages infected by *L. amazonensis* or *L. major* was evaluated using a degradative compartment marker, DQ-BSA (DQ^TM^ Red BSA; Molecular Probes, Eugene, OR, United States), as described by Lerena and Colombo (2011). CBA mouse macrophages were plated and, after 24 h, cells were washed and incubated with 10 μg/mL of DQ-BSA for 4 h at 37°C. Next, cells were rewashed and infected with metacyclic promastigotes of *L. amazonensis* or *L. major* labeled with CFSE (λ_ex_ 492 nm; λ_em_ 517 nm) at a ratio of 5:1 (parasite:macrophage) in accordance with the infection protocol (ii) described above in **Infection of macrophages with** ***L. amazonensis* or *L. major***. Following infection, cells infected for 4 h or more were incubated with 10 μg/mL rapamycin or 1 μM VPS34-IN1 (Bago et al., 2014), a specific PI3K class III inhibitor, for 2 h to evaluate the effect of autophagy modulation on DQ-BSA-labeled *Leishmania* parasitophorous vacuoles. Finally, the cells were fixed in 4% paraformaldehyde for 15 min and coverslips were mounted using a ProLong Gold Antifade kit containing DAPI (Life Technologies). Images were acquired via a Leica SP8 (Leica) confocal fluorescence microscope using a 63x/1.4 objective.

### LysoTracker Labeling of *L. amazonensis* or *L. major* Parasitophorous Vacuoles

LysoTracker^®^ Red DND-99 (Molecular Probes, Eugene, OR, United States), a weakly basic amine that selectively accumulates in cellular compartments with low internal pH, was used to characterize the parasitophorous vacuoles induced by *L. amazonensis* or *L. major* with respect to the acidity present in autophagosome compartments. Cells were labeled in accordance with a protocol previously described by Aguilera et al. (2009). CBA mouse macrophages were plated and infected as described above in the protocol (ii) of **Infection of macrophages with** ***L. amazonensis* or *L. major***. After 24 h of infection, macrophages were incubated with 100 nM of LysoTracker^®^ for 2 h. Cells were then fixed and coverslips were mounted as described in **Evaluation of hydrolytic activity in *L. amazonensis*- or *L. major*-induced parasitophorous vacuoles.** Images were acquired as described above.

### Evaluation of the Recruitment of LC3 to *L. amazonensis* or *L. major* Parasitophorous Vacuoles

Macrophages were infected as described above in the protocol (ii) of **Infection of macrophages with *L. amazonensis* or *L. major***. After infection procedures, labeling for the autophagic membrane marker LC3 was performed in accordance with the protocol described by Matte et al. (2016) using antibodies against LC3B (1:200) (NB600-1384, Novus Biologicals, Littleton, CO, United States), followed by AlexaFluor 488-conjugated goat anti-rabbit IgG (1:500) (ThermoScientific). Following infection, cells infected for 4 h or more were incubated with 10 μg/mL rapamycin or 1 μM VPS34-IN1 for 2 h to evaluate the effect of autophagy modulation on LC3-labeled *Leishmania* parasitophorous vacuoles. Cells were then fixed and mounted on coverslips as described above in **Evaluation of hydrolytic activity in *L. amazonensis*- or *L. major*-induced parasitophorous vacuoles.** Images were acquired as described above.

### Modulation of the Autophagic Process in *L. amazonensis* or *L. major* Infection

The effects of autophagy on *Leishmania* infection were assessed by quantifying the percentage of infected cells and the number of parasites per infected cell using optical fluorescence, or by determining the number of viable intracellular parasites by microscopy. For these assays, macrophages were first infected as described above in protocol (iii) of **Infection of macrophages with** ***L. amazonensis* or *L. major***.

To determine the percentage of infected cells and the number of parasites per infected cell, cells infected for 30 min were reincubated with either: (i) one of the following autophagic inhibitors: 10 μM chloroquine (Sigma, St Louis, MO, United States), 100 nM wortmannin (Sigma, St Louis, MO, United States) or 1 μM VPS34-IN1; (ii) or one of the following autophagic inducers: 10 μg/mL rapamycin (Sigma, St. Louis, MO, United States), or nutrient-poor EBSS medium (Sigma, St. Louis, MO, United States). After 4 h, all cells were washed and reincubated in complete DMEM for an additional 24 h. Cells were then subsequently fixed in 4% paraformaldehyde for 15 min and coverslips were mounted using a ProLong Gold Antifade kit with DAPI (Life Technologies). The percentage of infected cells and the number of parasites per infected macrophage were determined by counting no less than 400 cells in random fields under an Olympus BX 51 fluorescence microscope using an 100x/1.4 objective in accordance with a protocol previously described by Huynh et al. (2006).

Intracellular parasite viability was assessed by determining the number of viable intracellular amastigotes. After a 30-min infection period, cells were incubated with either one of the two autophagy inhibitors (10 μM chloroquine or 1 μM VPS34-IN1) or an autophagic inducer (10 μg/mL rapamycin) or by starvation, incubating cells in nutrient-poor medium (EBSS). After 4 h, all cells were washed and incubated in complete DMEM medium for 0, 6, or 12 h, followed by a 5-day period of reincubation in Schneider’s medium at 23°C. Finally, the viable intracellular amastigotes that had transformed into motile extracellular promastigotes were collected and counted in a Neubauer chamber.

As determined by AlamarBlue^®^ assays, the autophagy modulators employed in the present study did not present direct effects on axenic *Leishmania* viability at the concentrations employed in this study (data not shown).

### Quantification of Nitric Oxide (NO) Production and Arginase Activity

To determine whether autophagic modulation interferes with NO production by infected macrophages and/or arginase activity, cells were primed with 50 UI/mL IFN-γ (R&D Systems, Minneapolis, MN, United States) for 24 h and subsequently infected as described above in protocol (iii) of **Infection of macrophages with** ***L. amazonensis* or *L. major***. Next, macrophages were treated with 1 μM VPS34-IN1 or 10 μg/mL rapamycin. After 4 h, all cells were washed and reincubated in DMEM medium containing IFN-γ for an additional 24 h. Infected cells incubated in IFN-γ-free medium were used as a negative control. NO production was measured in culture supernatants by determining the accumulation of nitrite using the Griess reaction. Arginase activity was quantified by a colorimetric assay for the detection of urea as previously described (Abebe et al., 2013).

### Statistical Analysis

Graphing and statistical analysis were performed using the GraphPad Prism program (version Mac 5.0c). After verifying data normality, the unpaired Student’s *t-test* or the Mann–Whitney test were used for comparisons between two groups. For Gaussian distributions, one-way ANOVA was used, while for non-Gaussian distribution, the Kruskal–Wallis non-parametric test was employed for comparisons among three or more groups. Differences among groups were considered statistically significant when *p* < 0.05. Principal component analysis (PCA) and Hierarchical cluster analysis (Ward’s method) were used to test whether *L. amazonensis* and *L. major* could be clustered separately by the percentage of infected macrophages, parasite load, intracellular viability, percentage of LC3-decorated vacuoles as well as percentage of DQ-BSA and LysoTracker positives vacuoles. These data analyses were performed using JMP 11.0 (SAS) software.

## Results

### *L. amazonensis* and *L. major* Induce Autophagy in Infected Macrophages

**FIGURE 1:**
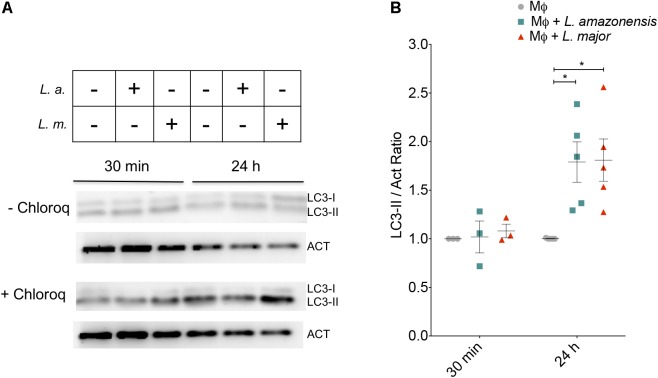
Autophagy is activated similarly in *L. amazonensis*- and *L. major*-infected cells. CBA mouse macrophages were infected with metacyclic promastigotes of *L. amazonensis or L. major*. **(A)** Western blot of LC3-II and Actin (Act) expression in CBA mouse macrophages infected with *L. amazonensis* or *L. major* for 30 min or for 4 h, followed by a 24 h-incubation period. Autophagic flux inhibition was achieved by treating the macrophages for 6 h with chloroquine (10 μM) in both uninfected and infected cells Actin was used as internal loading control. **(B)** Protein bands were densitometrically quantified to determined LC3II/Act ratios. Symbols represent individuals experiments, while lines are representative of means ± SE (One-way ANOVA, ^∗^*p* < 0.05). MΦ: Macrophages; MΦ + *L.a.*: Macrophages infected with *L. amazonensis*; MΦ + *L.m*.: Macrophages infected with *L. major.*

The LC3-II/Act ratio was employed as an approach to evaluate activation of the autophagic pathway. At 30 min of infection, no changes in the LC3-II/Act ratio were found under either *L. amazonensis* or *L. major* infection (**Figure 1**). After 24 h, similar increases in the LC3-II/Act ratio were seen in both *L. amazonensis*- and *L. major-*infected macrophages (1.79- and 1.80-fold, respectively) in comparison to uninfected macrophages (**Figure 1**). LC3-II/Act ratio was found to be higher in positive controls compared to control untreated macrophages: 2.1-fold in macrophages treated with rapamycin and 3.2-fold in cells incubated under nutritional stress conditions (data not shown). Autophagic flux was evaluated by comparing the LC3-II band intensity in cells treated with chloroquine that blocks autophagic flux. As shown in **Figure 1** in *L. amazonensis*- or *L. major*-infected cells the levels of LC3-II are increased in chloroquine treated cells, indicating that the autophagic flux was normal in the infected cells.

### *L. amazonensis*-Induced Parasitophorous Vacuoles Recruit More LC3 Than *L. major*

**FIGURE 2:**
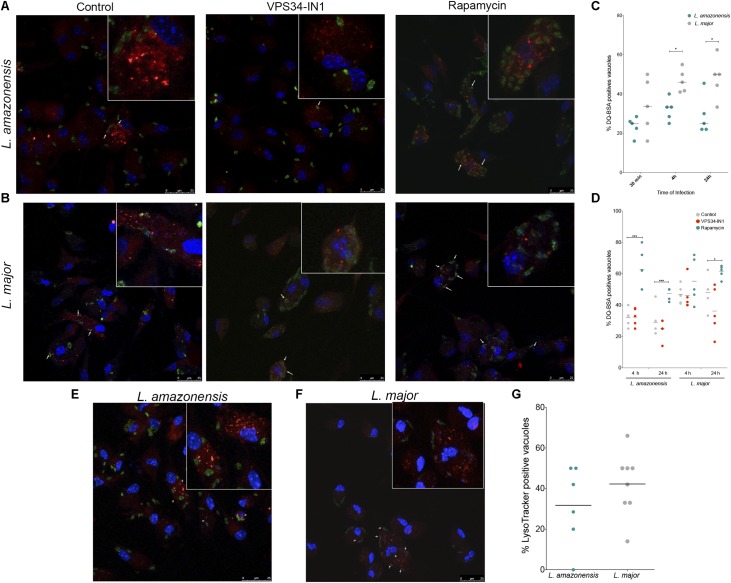
Characterization of autolysosomal features in parasitophorous vacuoles induced by *L. amazonensis* or *L. major*. **(A–D)** DQ-BSA labeling: CBA mouse macrophages were incubated with DQ-BSA (red), infected with metacyclic promastigotes of CFSE-labeled *L. amazonensis* or *L. major*, and then fixed. Groups of positive and negative control cells were incubated with 10 μg/mL rapamycin or 1 μM VPS34-IN1 after infection and then fixed. For confocal microscopy, cell nuclei were labeled with DAPI (blue). **(A)** *L. amazonensis*- (green) or **(B)** *L. major*- (green) infected macrophages labeled with DQ-BSA (red). **(C)** Percentage of *L. amazonensis* and *L. major*-induced parasitophorous vacuoles labeled with DQ-BSA (Student’s *t*-test, ^∗^*p* < 0.05). **(D)** Percentage of DQ-BSA-labeled *Leishmania-*induced vacuoles in macrophages treated with VPS34-IN1 or rapamycin, or in untreated control cells (One-way ANOVA, ^∗∗∗^*p* < 0.01). **(E–G)** LysoTracker labeling: Macrophages were infected with metacyclic promastigotes of CFSE-labeled *L. amazonensis* or *L. major* and labeled with LysoTracker (100 nM) followed by fixing. For confocal microscopy, cell nuclei were labeled with DAPI (blue). **(E)** *L. amazonensis*- (green) and **(F)** *L. major*-infected macrophages (green) labeled with LysoTracker (red). **(G)** Percentage of *L. amazonensis-* and *L. major*-induced parasitophorous vacuoles labeled with LysoTracker (Student’s *t*-test, ^∗^*p* > 0.05). White arrows indicate labeled parasitophorous vacuoles induced by *Leishmania*, and circles correspond to each randomly selected field analyzed.

The characteristics of parasitophorous vacuoles were evaluated to determine whether dissimilarities in the capacity of *L. amazonensis*- and *L. major*-induced parasitophorous vacuoles to interact with the autophagic pathway can account to differences in parasite survival inside host cells (Alexander and Vickerman, 1975; Antoine et al., 1998; Wilson et al., 2008; Real and Mortara, 2012). First, the hydrolytic activity inside these compartments was assessed by determining the percentage of parasitophorous vacuoles labeled with DQ-BSA. After 30 min of infection, the percentage of *L. amazonensis*- and *L. major*-induced parasitophorous vacuoles labeled with DQ-BSA was similar (**Figures 2A–C**). By contrast, after 4 h of infection, *L. major* vacuoles (46%) exhibited greater positivity for DQ-BSA than those induced by *L. amazonensis* (33.33%) (**Figure 2C**). Similarly, after 24 h of infection, 50% of the *L. major*-induced parasitophorous vacuoles were positive for DQ-BSA versus only 25% of the *L. amazonensis* vacuoles (*p* < 0.01, **Figure 2C**). The positivity in DQ-BSA was enhanced by 27.58% for *L. amazonensis*- and 57.33% for *L. major*-induced parasitophorous vacuoles in macrophages in which autophagy was induced by rapamycin, as compared to untreated cells (*p* < 0.01, **Figure 2D**). In spite of this, DQ-BSA positivity was statistically similar in both the vacuoles induced by *L. amazonensis* and *L. major* (*p* > 0.05, **Figure 2D**), which indicates that *L. amazonensis*- and *L. major*-induced parasitophorous vacuoles similarly acquire degradative features in macrophages in which autophagy was induced via mTOR inhibition by rapamycin. In addition, LysoTracker labeling after 24 h of infection revealed that the percentages of parasitophorous vacuoles that accumulated this fluorescent probe are different in *L. major*-infected cells (42.25%) and *L. amazonensis*-infected macrophages (31.75), although this difference is not statistically significant (*p* > 0.05, **Figures 2E–G**).

**FIGURE 3:**
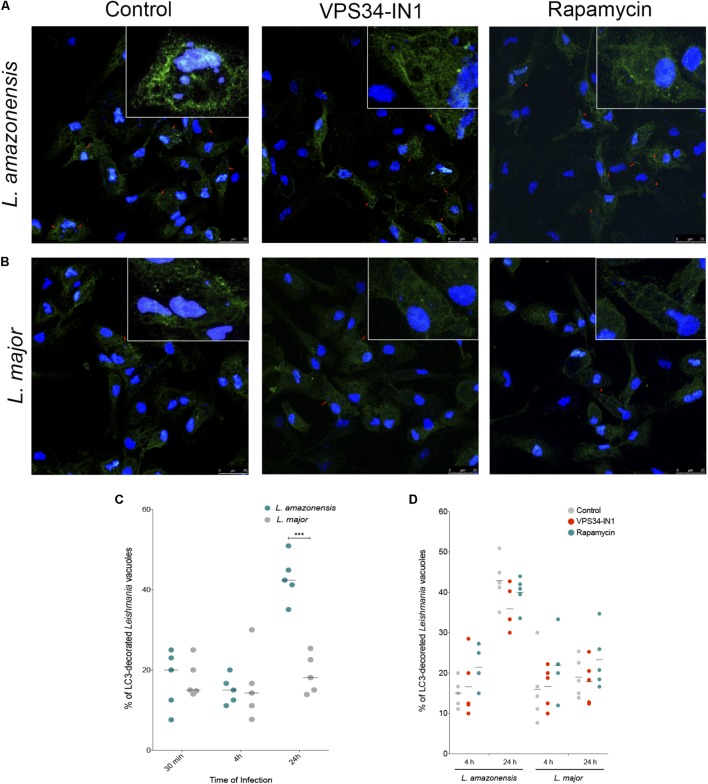
Assessment of LC3 recruitment to parasitophorous vacuoles induced by *L. amazonensis* and *L. major*. CBA mouse macrophages were infected with metacyclic promastigotes of *L. major* or *L. amazonensis*. A group of control cells were incubated with 10 μg/mL rapamycin or 1 μM VPS34-IN1 or left untreated after infection. Cells were then fixed and labeled with anti-LC3 antibody followed by the secondary anti-rabbit IgG antibody conjugated to Alexa Fluor 488 (green). For confocal microscopy, cell nuclei were labeled with DAPI (blue). Confocal microscopy images of **(A)** *L. amazonensis*- or **(B)** *L. major*-infected macrophages labeled with LC3. **(C)** Percentage of *L. amazonensis-* or *L. major*-induced parasitophorous vacuoles decorated with LC3-II (Student’s *t*-test, ^∗∗∗^*p* < 0.01). **(D)** Percentage of LC3-decorated *Leishmania-*induced vacuoles in macrophages treated with VPS34-IN1 or rapamycin, or in untreated control cells (One-way ANOVA, *p >* 0.05). Circles correspond to each randomly selected field analyzed.

LC3 labeling showed positivity even at early stages of infection, i.e., 30 min, as *L. amazonensis-* and *L. major-*induced parasitophorous vacuoles were decorated with endogenous LC3. In fact, at 30 min and 4 h after infection, both *L. amazonensis*- and *L. major*-infected macrophages exhibited similar percentages of LC3 decorated parasitophorous vacuoles (**Figures 3A–C**). Only after 24 h of infection differences were observed in endogenous LC3 labeling in these compartments, with 42.36% of positivity detected in *L. amazonensis*-induced parasitophorous vacuoles versus only 18.10% in *L. major* (*p* < 0.01, **Figure 3C**).

Additional control experiments using macrophages in which autophagy was inhibited by VPS34-IN1, a specific PI3K class III inhibitor, or induced by rapamycin showed no alterations in LC3 positivity as measured by the percentage of *L. amazonensis-* or *L. major-*induced vacuoles decorated with LC3 in comparison to infected untreated macrophages (**Figure 3D**). This result indicates that LC3-recruiment to *Leishmania*-induced parasitophorous vacuoles seems to be independent of the PI3k-Akt-mTOR pathway.

### Autophagy Induction Favors *L. amazonensis* and *L. major* Intracellular Viability

**FIGURE 4:**
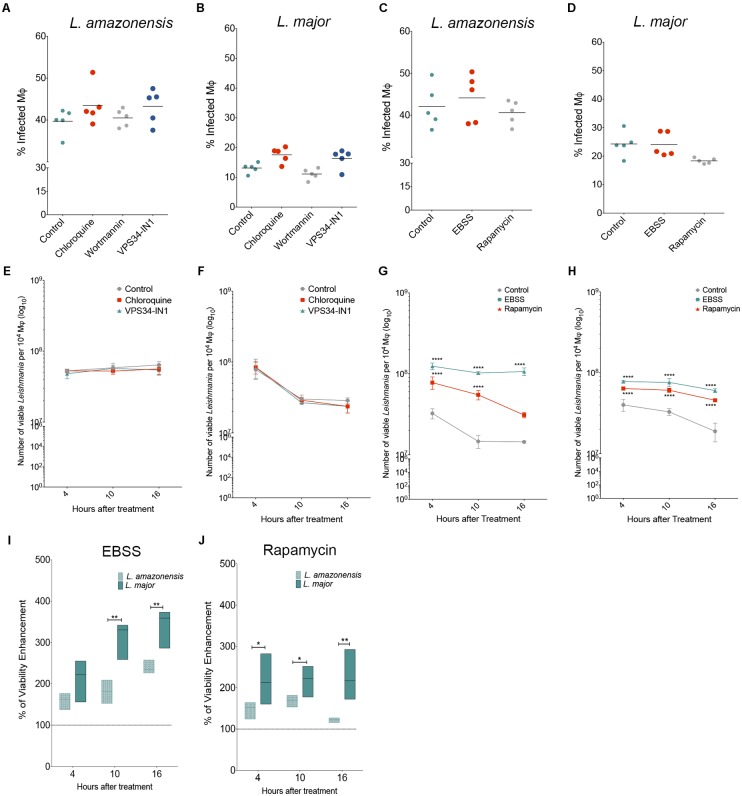
Effect of autophagy modulation on *Leishmania* infection. CBA mouse macrophages were infected with *L. amazonensis* or *L. major* and incubated with different autophagic modulators. **(A–D)** To determine the percentage of *L. amazonensis*- or *L. major*-infected cells were incubated with autophagic inhibitors **(A,B)**: chloroquine (10 μM), wortmannin (100 nM) or VPS34-IN1 (1 μM), or with autophagic inducers **(C,D)**: nutrient-poor EBSS medium or rapamycin (10 μg/mL). After 4 h, all cell groups were reincubated for an additional 24 h, fixed and stained with DAPI. Circles represent data from each replicate of one of two independent experiments performed in quintuplicate (Kruskal–Wallis test, Dunns post-test, *p* > 0.5). **(E–J)** To assess intracellular **(E,G)** *L. amazonensis* or **(F,H)** *L. major* viability, infected macrophages were incubated with the **(E,F)** autophagic inhibitors: chloroquine (10 μM) or VPS34-IN1 (1 μM) or **(G,H)** autophagic inducers: nutrient-poor EBSS medium or rapamycin (10 μg/mL). After incubation, cells were reincubated in Schneider’s medium to determine the number of viable parasites. **(I,J)** Percentage of enhancement in intracellular parasite viability after incubation with **(I)** EBSS or **(J)** rapamycin. Circles correspond to the mean of one representative out of two independent experiments performed in quintuplicate ± SD (Two-Way ANOVA test, Tukey’s multiple comparisons post-test ^∗∗∗∗^*p* < 0.0001). Lines represent median values and floating bars show quartiles (25% and 75%) of one representative experiment out of two independent experiments performed in quintuplicate (Mann–Whitney Test, ^∗^*p* < 0.05 and ^∗∗^*p* < 0.01).

The role played by autophagy in *Leishmania* infection was evaluated using autophagic modulators, followed by the determination of the percentage of infected macrophages and number of viable intracellular parasites. No alterations were seen in the percentage of macrophages infected with *L. amazonensis* or *L. major* (**Figures 4A–D**) or in the numbers of *Leishmania* per infected cell (**Supplementary Figure S1**) in comparison to untreated macrophages, regardless of autophagic inhibition by chloroquine, wortmannin or VPS34-IN1or induction by rapamycin or starvation. Also, none of the autophagic inhibitors influenced the number of viable intracellular *L. amazonensis* or *L. major* parasites (**Figures 4E,F**). However, we observed that induction by starvation or treatment with rapamycin enhanced the number of recovered viable parasites of both *L. major* and *L. amazonensis* species in comparison to infected untreated controls (**Figures 4G,H**). Pharmacologically induced autophagy by rapamycin led to significant increases of 140.25, 146.18, and 157.15% in the number of viable *L. major* intracellular parasites after 4, 10, and 16 h of infection, respectively, in comparison to controls (**Figure 4H**) for *L. amazonensis*, differences of 61.7 and 76.39% were seen in the number of viable parasites at 4 and 10 h after treatment, respectively (**Figure 4G**). Interestingly, we observed that the physiological induction of autophagy by starvation or pharmacological induction by rapamycin led to a greater enhancement in *L. major* intracellular parasite viability than *L. amazonensis* in comparison to untreated infected cells (**Figures 4I,J**). The physiological induction of autophagy was shown to enhance intracellular viability by 2.5 and 1.5 times in *L. major* versus *L. amazonensis*, respectively, at 10 and 16 h after treatment (*p* < 0.05) (**Figure 4I**). Additionally, the pharmacological induction of autophagy by rapamycin enhanced *L. major* intracellular viability by 2.17 times as compared to *L. amazonensis* viability at as early as 4 h after treatment (*p* < 0.05) (**Figure 4J**). Additionally, similar enhancements in intracellular parasite viability were seen in *L. major* compared to *L. amazonensis* at both 10 and 16 h after treatment (**Figure 4J**).

### Autophagic Induction Reduces NO Production by *L. major*- and *L. amazonensis*-Infected Macrophages

**FIGURE 5:**
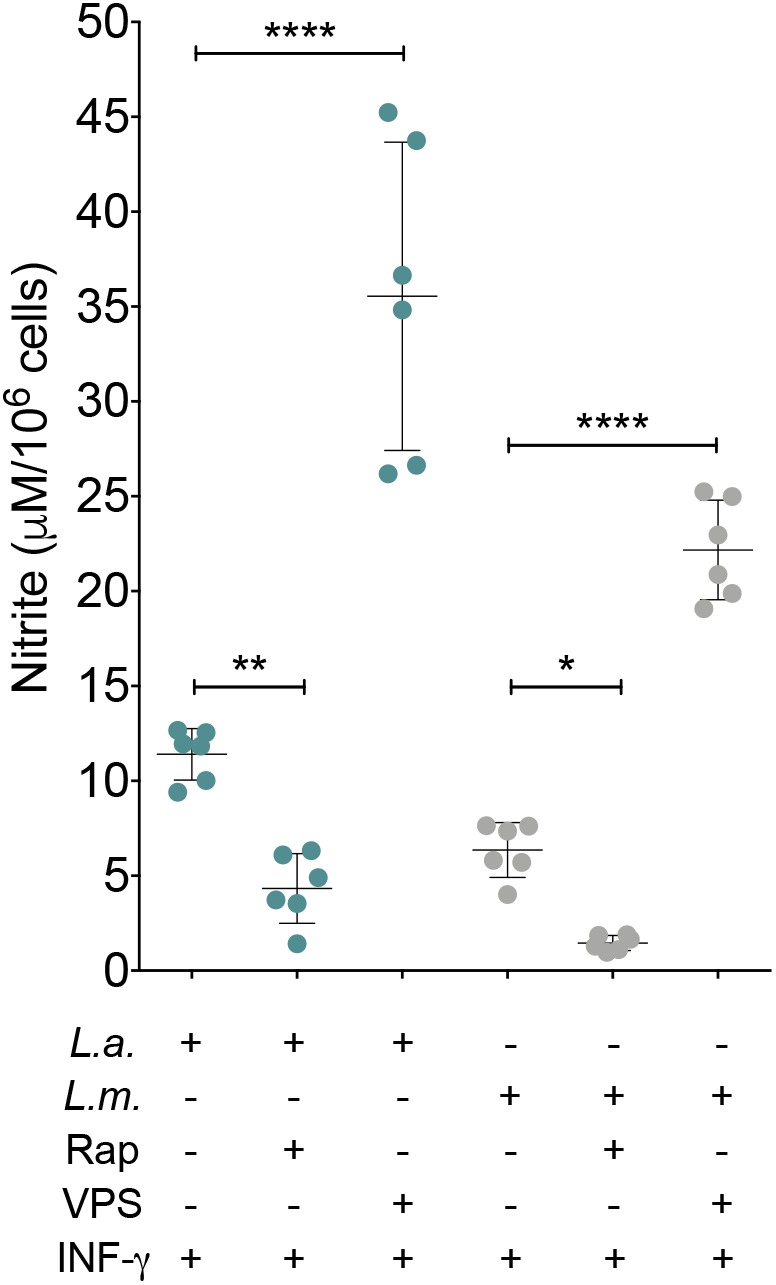
Autophagic induction reduces NO production by *L. major*- and *L. amazonensis*-infected macrophages. CBA mouse macrophages were infected with *L. amazonensis* or *L. major* and incubated with VPS34-IN1 (1 μM) or rapamycin (10 μg/mL). After 4 h, all cell groups were reincubated in modulator-free medium for an additional 24 h. Macrophage NO production was measured by detecting nitrite in culture supernatants. Circles correspond to each replica of one representative of two independent experiments performed in sextuplicate ± SD (One-way ANOVA, ^∗^*p* < 0.05, ^∗∗^*p* < 0.01 and ^∗∗∗∗^*p* < 0.0001). *L.a.*, *L. amazonensis*; *L.m*., *L. major*; Rap, rapamycin; VPS, VPS34-IN1.

To determine whether NO and arginase are involved in the enhanced *L. amazonensis* and *L. major* intracellular viability induced by autophagy, we evaluated the effect of rapamycin-induced autophagy on NO production and arginase activity in infected macrophages. Unstimulated control cells, both uninfected and infected with *L. amazonensis* or *L. major*, produced NO at undetectable levels. Uninfected macrophages stimulated with IFN-γ released 2.30 μM of NO in the culture medium (data not shown). The addition of rapamycin or VPS24-IN1 to these cell cultures altered NO production to 0.74 or 4.61 μM, respectively (data not shown). The addition of rapamycin to *L. amazonensis*-infected macrophages resulted in a NO production level of 4.33 μM in comparison to controls (11.40 μM) (*p* < 0.05, **Figure 5**). Additionally, VPS34-IN1 enhanced NO production by *L.* amazonensis-infected macrophages (35.55 μM) in comparison to control cells (11.40 μM) (*p* < 0.05, **Figure 5**). Similarly, in *L. major*-infected macrophages, the addition of VPS34-IN1 to cell cultures increased NO levels to 22.17 μM, while the addition of rapamycin reduced NO production to 1.46 μM in comparison to 6.35 μM of NO released by untreated *L. major*-infected macrophages (*p* < 0.05, **Figure 5**). Interestingly, no significant differences were observed in arginase activity between the *Leishmania*-infected macrophages treated with rapamycin or VPS34-IN1 and untreated control macrophages (*p* > 0.05, **Supplementary Figure S2**). This seems to suggest that the induction of autophagy favors *Leishmania* intracellular viability by reducing NO production.

### Autophagic Markers Can Discriminate *L. amazonensis*- and *L. major*-Infected Macrophages

**FIGURE 6:**
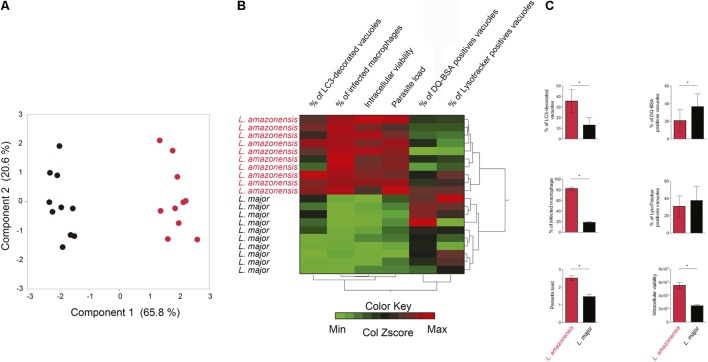
*L. amazonensis*- and *L. major-*infected macrophages are clustered in different groups regarding autophagic features and infection parameters. Cluster analysis was performed using the following variables: percentage of infected macrophages, intracellular viability, number of *Leishmania* per infected macrophages, percentage of LC3 decorated *Leishmania* vacuoles and percentage of DQ BSA and LysoTracker positives vacuoles. **(A)** Principal component analysis. Black dots represent *L. major*-infected macrophages and red dots *L. amazonensis-*infected macrophages. **(B)** Hierarchical cluster analysis (Ward’s method). Red represents maximum in the color key, green represents minimum and mean values are shown in black. **(C)** Statistical analysis of autophagic features and infection parameters of *L. amazonensis-* and *L. major*-infected cells evaluated in the heat map (Student’s *t* Test, ^∗^*p* < 0.05).

A PCA was used to verify whether the parameters above evaluated: characterization of parasitophorous vacuoles regarding autophagic features and the percentage of infected macrophages, parasitic load and intracellular parasite viability, could discriminate *L. amazonensis* and *L. major* infection (**Figures 6A–C**). The analyzed parameters allowed differentiation of two major groups separating the *L. amazonensis-*infected macrophages from those infected by *L. major* (**Figures 6A,B**). Additionally, heatmap analysis revealed that *L. amazonensis*-infected macrophages are grouped into a cluster associated to infected cells presenting higher percentage of LC3-decorated vacuoles, higher percentage of infected macrophages and higher intracellular parasite viability, while *L. major*-infected macrophages clustered in a group associated to higher percentage of vacuoles positive for DQ-BSA and LysoTracker (**Figure 6B**).

## Discussion

The present study demonstrated that *L. amazonensis* and *L. major* both induce autophagy in CBA macrophages, yet *L. amazonensis* infection was shown to produce a higher percentage of parasite-induced vacuoles decorated by endogenous LC3-II. On the other hand, the compartments induced by *L. amazonensis* presented lower positivity for the degradative marker DQ-BSA. With respect to autophagy modulation in *L. amazonensis* and *L. major* infection *in vitro*, no changes were seen in the percentage of *L. amazonensis* or *L. major* infected cells or parasite load when autophagy was induced or inhibited, either physiologically or pharmacologically. While the inhibition of autophagy did not affect *Leishmania* intracellular viability, exogenously induced autophagy was shown to enhance intracellular parasite viability.

The present study also found comparatively similar increases in the LC3-II/Act ratio in inflammatory peritoneal CBA mouse macrophages infected with *L. amazonensis* or *L. major*. We further demonstrated that increased levels of LC3-II were associated with the induction of autophagy *per se*, i.e., not via the blockage of autophagic flux, since lower levels of LC3-II were seen in infected cells compared to those treated with chloroquine, an inhibitor of autophagic flux. These findings stand in agreement with previous studies reporting increased LC3 expression in RAW cells, BALB/c and C57BL/6 bone marrow macrophages infected by *L. amazonensis* (Cyrino et al., 2012) or *L. major* (Franco et al., 2017), and BALB/c bone marrow macrophages infected with *L. major* (Frank et al., 2015) in comparison to uninfected cells. Consistent with our results and those reported in other *in vitro* studies, Mitroulis et al. (2009) observed a greater conversion of LC3-I to LC3-II in a sample of bone marrow macrophages from a male patient with visceral leishmaniasis infected with *L. donovani* in comparison to a bone marrow sample from a healthy patient. These findings seem to suggest that, regardless of species, *Leishmania* infection provokes the activation of the autophagic pathway in host cells *in vitro* and *in vivo*.

Several studies have successfully demonstrated that vacuoles induced by intracellular microorganisms can be loaded with DQ-BSA or LysoTracker (Gutierrez et al., 2004; Aguilera et al., 2009; Lerena and Colombo, 2011). While these soluble markers are not specific to autophagy, their use in labeling in conjunction with other autophagic compartment markers represents a suitable and reliable tool to monitor acidity and degradative activity in compartments, such as autolysosomes (Klionsky et al., 2007; Mitou et al., 2009; Vazquez and Colombo, 2009). Our analysis found no significant differences in the percentages of LysoTracker-labeled vacuoles induced by either *L. amazonensis* or *L. major*. Labeling of both *L. amazonensis*- and *L. major*-induced parasitophorous vacuoles with this lysosomal dye was previously demonstrated by Real and Mortara (2012). While these authors did not quantify the percentage of *Leishmania*-induced vacuoles labeled with LysoTracker, comparisons of the intensity of this probe revealed that the parasitophorous vacuoles induced by *L. major* presented lower intensity than *L. amazonensis*-induced compartments (Real and Mortara, 2012) suggesting that the acidity of these compartments was somehow diminished. In the present report, a higher percentage of DQ-BSA positivity was seen in *L. major*-induced vacuoles compared to those induced by *L. amazonensis* at both 4 and 24 h after infection. Previously, Beron et al. (2002) and Aguilera et al. (2009) successfully marked the large vacuoles induced by *Coxiella burnetti* with DQ-BSA. These vacuoles have been described as presenting morphological similarities to those induced by *L. amazonensis* (Antoine et al., 1990; Maurin et al., 1992; Veras et al., 1994, 1995). However, different from *C. burnetii*, *L. amazonensis* at later infection times maybe somehow preventing fusion with degradative compartments.

The recruitment of LC3 to *L. major*-induced parasitophorous vacuoles has been previously demonstrated (Crauwels et al., 2015; Matte et al., 2016). Crauwels et al. (2015) also reported greater percentages (92 ± 0.7%) of LC3-positive parasitophorous vacuoles induced by apoptotic *L. major* in comparison to those induced by viable *L. major* (7 ± 1%). Herein, we found similar percentages of LC3-decorated membranes of both *L. amazonensis*- and *L. major*-induced parasitophorous vacuoles as early as 30 min after infection. However, after 24 h, greater LC3-positivity was detected in *L. amazonensis*-induced parasitophorous vacuoles than in those induced by *L. major*. Interestingly, no changes in the percentage of LC3 positivity were seen in the vacuoles induced by *L. amazonensis* or *L. major* in macrophages treated with VPS34-IN1 or rapamycin when compared to untreated control macrophages. A similar result was described by Thomas et al. (2017), who found no differences in LC3-II levels between *L. donovani*-infected human THP-1 cells and those infected and treated with rapamycin. These authors hypothesized that the lack of an increase in LC3-II positivity observed in *L. donovani*-infected cells previously treated with rapamycin could be due to the fact that this parasite species inhibits classical autophagy activation via the PI3k-Akt-mTOR pathway, instead inducing this process via another pathway independent of mTOR. Accordingly, it is possible that in our CBA mouse macrophage model, this same mTOR-independent pathway was used to activate autophagy by both *L. amazonensis* and *L. major*. Alternatively, it is plausible to suggest that treatment with rapamycin resulted in a lack of enhancement in the percentage of LC3-II-decorated vacuoles in *Leishmania*-infected macrophages, associated with the more intense DQ-BSA labeling also found in *Leishmania* vacuoles, could be a result of the previously described accumulation of hydrolytic enzymes and, subsequently, increased cleavage of LC3-II in the inner membrane of these compartments (Kirisako et al., 2000; Tanida et al., 2004).

Many studies have been carried out in an attempt to determine whether parasite survival is favored by host cell autophagy under infection by different *Leishmania* species, or whether it functions as a defense mechanism. Of note, PCA and heat map analyses performed in the present study, completely discriminated *L. major*-infected macrophages, which grouped for higher positivity for markers of degradative compartments, DQ-BSA and Lysotracker, from *L. amazonensis*-infected cells that clustered for higher intensity of infection and LC3 positivity. Unexpectedly, autophagic inhibition exerted no effects on *Leishmania* spp infection, while exogenously induced autophagy was shown to favor intracellular viability of both parasites, although in a higher extent for *L. major* than *L. amazonensis*. In conjunction, these findings suggest that triggering autophagic process by PI3k-Akt-mTOR pathway seems to be beneficial to *Leishmania* spp intracellular survival, more intensely for those species that survive in a compartment presenting degradative characteristics. Thus, autophagy induction after infection would be detrimental to the host, since it would favor the intracellular viability of *Leishmania*. Consistently with our findings, Pinheiro et al. (2009) demonstrated that autophagy induced by starvation after infection led to an increase in the intracellular viability of *L. amazonensis* in BALB/c and A/J macrophages, as well as in the J774 cell-line. However, in contrast to our findings, these same authors found that starvation-induced autophagy did not alter the intracellular viability of *L. amazonensis* in infected C57BL/6 macrophages or that of *L. major* in infected BALB/c macrophages (Pinheiro et al., 2009). Moreover, it has also been demonstrated that pre-infection autophagic inhibition via Atg5 knockdown in bone-marrow macrophages resulted in an increase in *L. major* parasite burden (Frank et al., 2015). In the present study, autophagy has been modulated after infection, since, previously, we found that the induction of autophagy prior to infection inhibited the phagocytic capacity of mammalian cells in a non-specific manner (Lima et al., 2011). It is possible that the reduction in Atg5 expression induced by Frank et al. (2015) in bone marrow macrophages provoked an enhancement in *L. major* infection due to an increase in parasite uptake, as previously described for *M. tuberculosis* (Bonilla et al., 2013). Accordingly, we propose that results reported by these previous studies, which sought to assess the effects of knocking down autophagy-related genes in the context of *Leishmania* infection, serve to reinforce the notion that inhibiting or inducing autophagy prior to infection profoundly interferes with phagocytic capacity of cell, rather than affecting the intracellular survival of pathogens.

By investigating how the exogenous induction of autophagy favors *Leishmania* intracellular viability, we have demonstrated that rapamycin decreases NO levels but does not significantly alter arginase activity. Consistent with our finding, Pinheiro et al. (2009) previously demonstrated that starvation-induced autophagy decreased NO production by *L. amazonensis*-infected macrophages in association with enhanced parasite intracellular viability. This mechanism seems to be universal, since it has already been shown that autophagic induction reduces NO generation by microglia (Han et al., 2013), the RAW 264.7 macrophage cell line (Jin et al., 2009), and also by ischaemic/reperfused myocardium (Ma et al., 2018). We speculate that, in our model, rapamycin may enhance parasite viability via reduced NO production, by way of one of the two following mechanisms: the suppression of mRNA expression of inducible nitric oxide synthase (iNOS) (Han et al., 2013) or the induction of iNOS proteasomal degradation (Jin et al., 2009).

Taken together, the present findings seem to indicate that although both of the evaluated species of *Leishmania* activate the autophagic pathway similarly in CBA mouse macrophages, the resulting parasitophorous vacuoles present different characteristics: enhanced LC3 recruitment is observed in *L. amazonensis*-induced parasitophorous vacuoles in comparison to *L. major*-induced vacuoles, which present more degradative features (result PCA). Despite these differences, we demonstrated that autophagic induction favors intracellular parasite survival in both *L. amazonensis* and *L. major*, albeit more pronounced in *L. major*, which seems to be related to decreased NO production. Alternatively, it is possible that autophagosomes may provide metabolites and amino acids to *L. major*- and *L. amazonensis*-induced vacuoles, which can favor the growth and intracellular development of *Leishmania.* One of these metabolites may be iron, since stored within ferritin, can be mobilized and released through autophagy (Linder, 2013; Niu et al., 2016) and then transported into the leishmanial cytosol by LIT1 (Huynh et al., 2006).

## Author Contributions

BD and PV conceived and designed the experiments. BD, CS, JL, NJA, TS, and JF-C performed the experiments. BD, KF, CB, JM, MC, and PV analyzed the data. PV contributed reagents, materials, and analysis tools. BD and PV wrote the manuscript. All the authors contributed to manuscript elaboration and revision and approved the final version prior to submission.

## Conflict of Interest Statement

The authors declare that the research was conducted in the absence of any commercial or financial relationships that could be construed as a potential conflict of interest.

## Supplementary Material

FIGURE S1 Effect of autophagic modulation on the number of *Leishmania* per infected macrophage. CBA mouse macrophages were infected with *L. amazonensis* or *L. major* and incubated with different autophagic modulators. To determine parasite load, *L. amazonensis* or *L. major*-infected cells were incubated with **(A)** autophagic inhibitors: chloroquine (10 μM), wortmannin (100 nM) or VPS34-IN1 (1 μM), or with **(B)** autophagic inducers: nutrient-poor EBSS medium or rapamycin (10 μg/mL). After 4 h, all cell groups were reincubated for an additional 24 h, fixed and stained with DAPI. Circles represent data from each replicate of one of two independent experiments performed in quintuplicate (Kruskal–Wallis test, Dunns post-test, *p* > 0.5).

FIGURE S2 Effect of autophagic modulation on arginase activity. CBA mouse macrophages were infected with *L. amazonensis* or *L. major* and incubated with VPS34-IN1 (1 μM) or rapamycin (10 μg/mL). After 4 h, all cell groups were reincubated in modulator-free medium for an additional 24 h. Arginase activity was determined by urea production assay. Circles correspond to each replica of one experiment performed in sextuplicate ± SD (One-way ANOVA). *L.a.*, *L. amazonensis*; *L.m*., *L. major*; Rap, rapamycin; VPS, VPS34-IN1.
