# Supplementary figures and images for: Autophagic Induction Greatly Enhances Leishmania major Intracellular Survival Compared to Leishmania amazonensis in CBA/j-Infected Macrophages
Source: Front Microbiol. 2018 Aug 15;9:1890. doi: 10.3389/fmicb.2018.01890 (PMC6104192; doi:10.3389/fmicb.2018.01890)

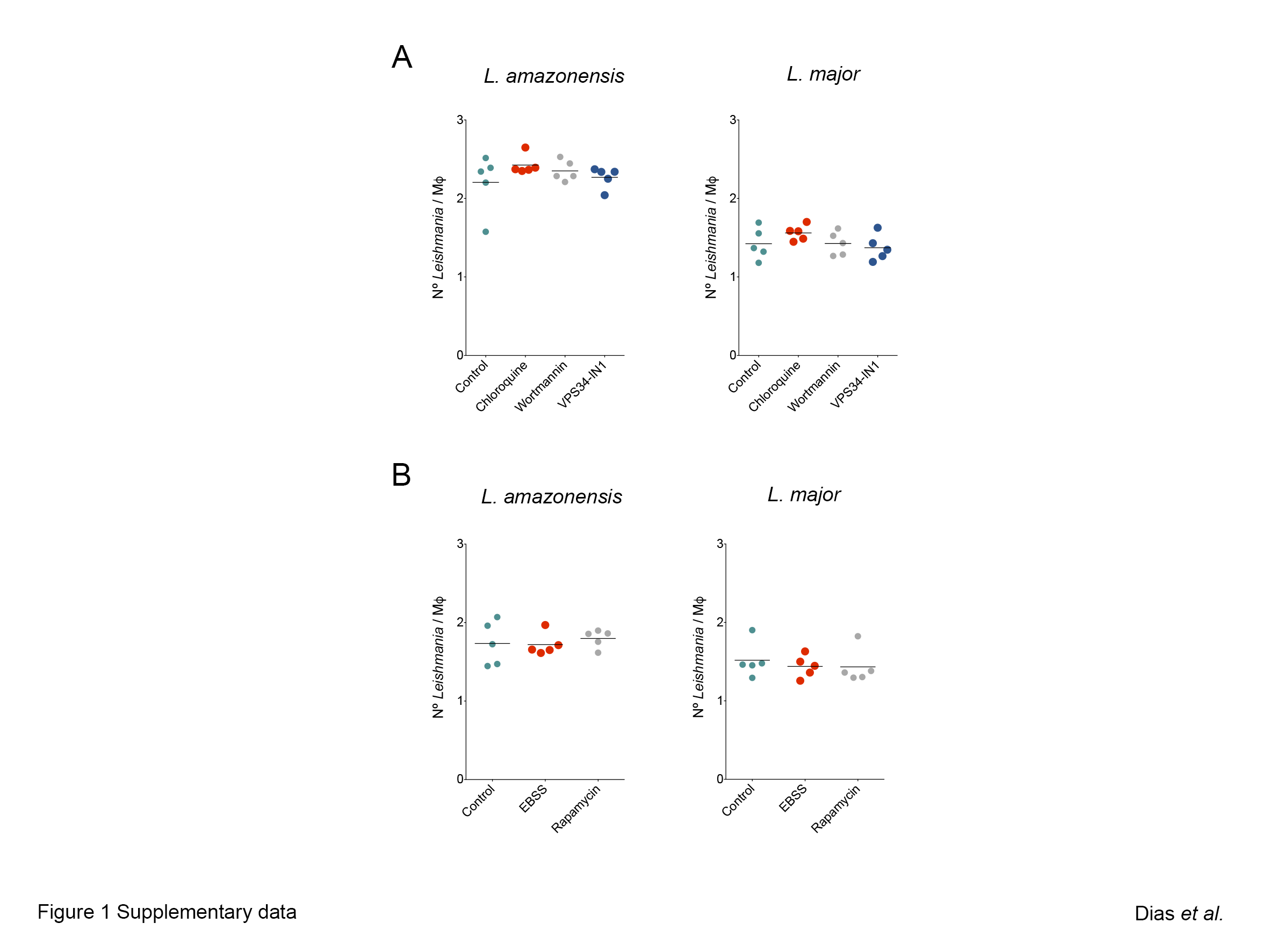

Supplement: FIGURE S1 — Effect of autophagic modulation on the number of Leishmania per infected macrophage. CBA mouse macrophages were infected with L. amazonensis or L. major and incubated with different autophagic modulators. To determine parasite load, L. amazonensis or L. major-infected cells were incubated with (A) autophagic inhibitors: chloroquine (10 μM), wortmannin (100 nM) or VPS34-IN1 (1 μM), or with (B) autophagic inducers: nutrient-poor EBSS medium or rapamycin (10 μg/mL). After 4 h, all cell groups were reincubated for an additional 24 h, fixed and stained with DAPI. Circles represent data from each replicate of one of two independent experiments performed in quintuplicate (Kruskal–Wallis test, Dunns post-test, p > 0.5). [file Image_1.TIF]

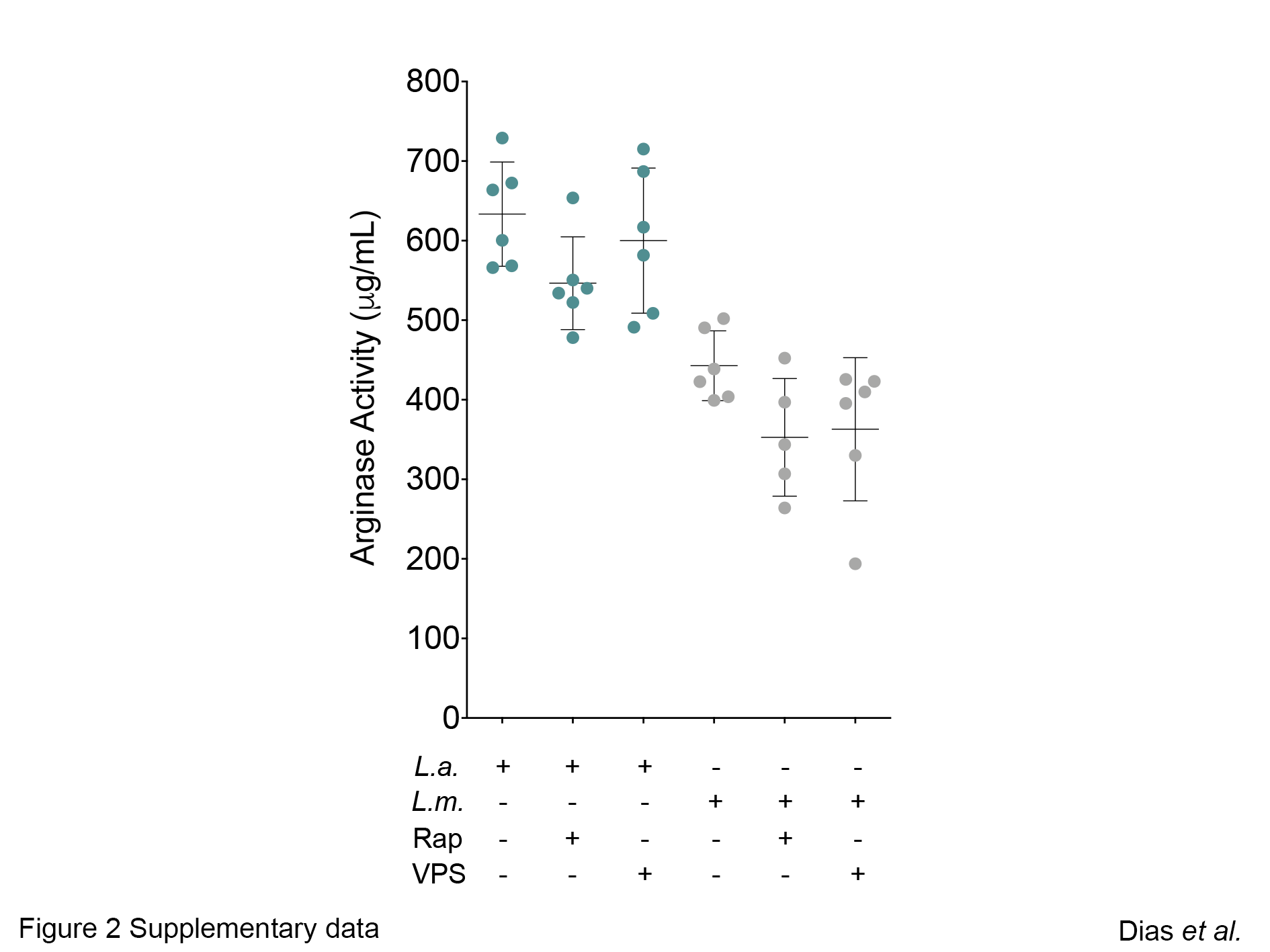

Supplement: FIGURE S2 — Effect of autophagic modulation on arginase activity. CBA mouse macrophages were infected with L. amazonensis or L. major and incubated with VPS34-IN1 (1 μM) or rapamycin (10 μg/mL). After 4 h, all cell groups were reincubated in modulator-free medium for an additional 24 h. Arginase activity was determined by urea production assay. Circles correspond to each replica of one experiment performed in sextuplicate ± SD (One-way ANOVA). L.a., L. amazonensis; L.m., L. major; Rap, rapamycin; VPS, VPS34-IN1. [file Image_2.TIF]
